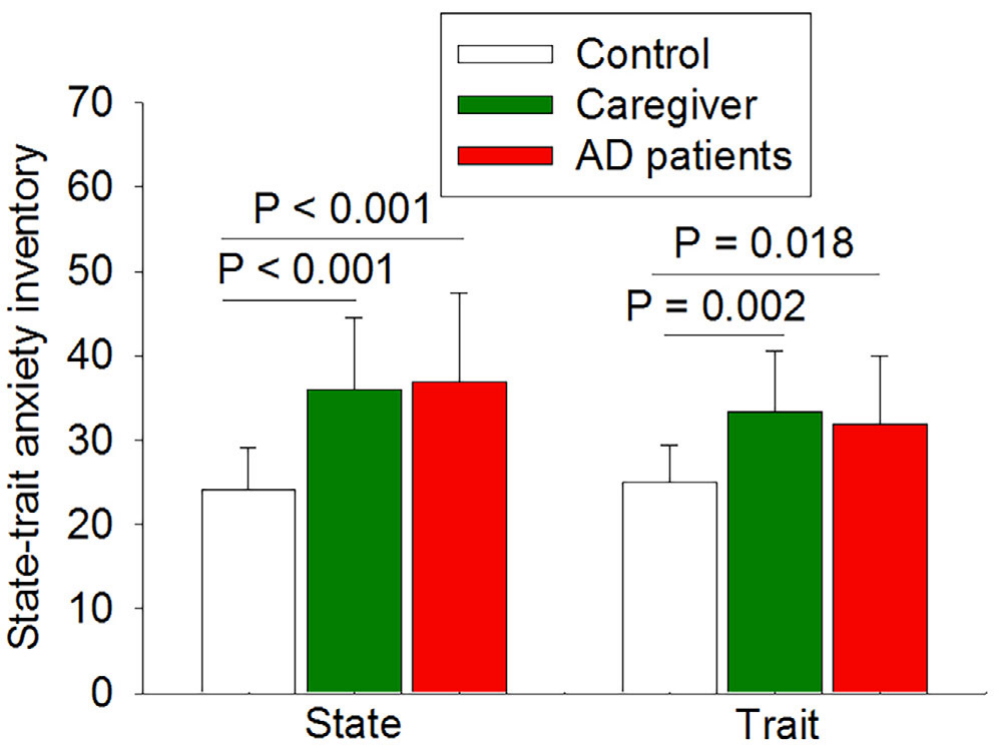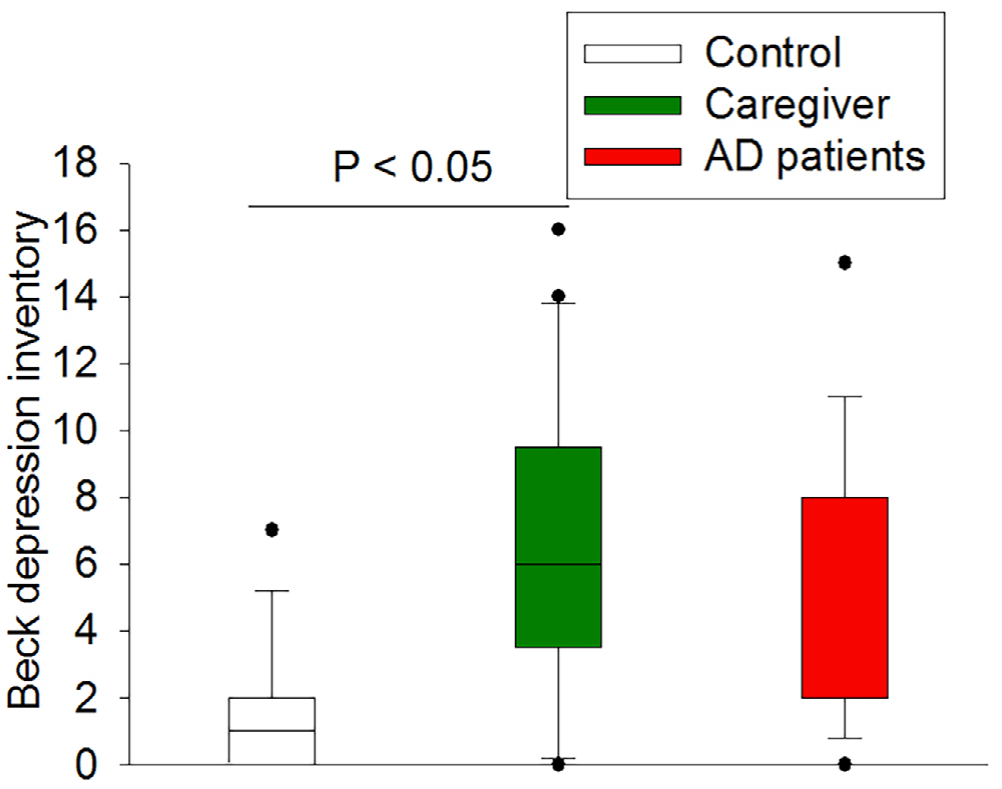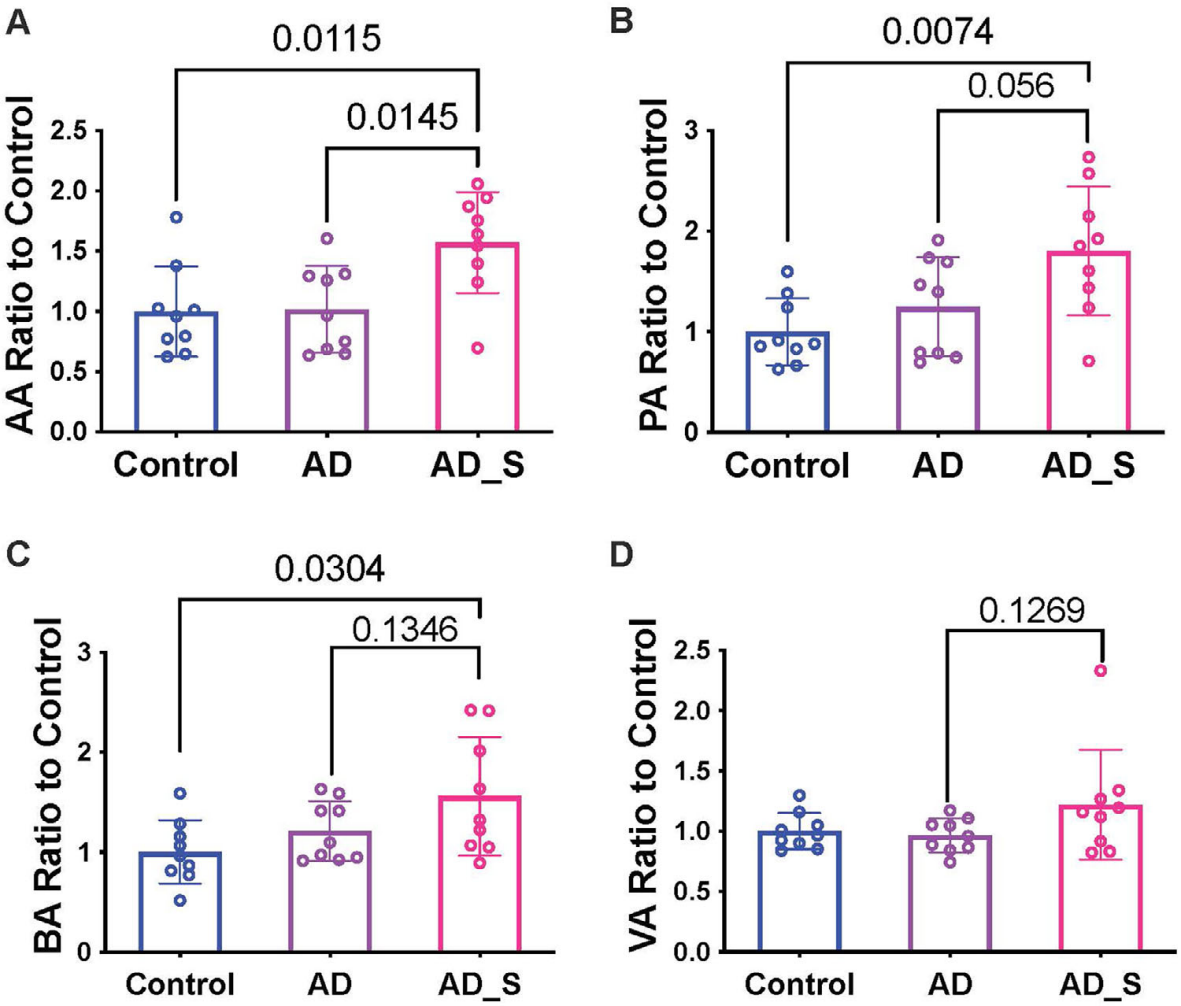# Spousal care partners have increased levels of anxiety, depression and blood short chain fatty acids

**DOI:** 10.1002/alz.086475

**Published:** 2025-01-09

**Authors:** Zhiyi Zuo, Zunaira Arbab, Weiran Shan, Avani Jain, Virginia Gallagher, Shannon Reilly, Anelyssa D’Abreu, Carol A Manning

**Affiliations:** ^1^ University of Virginia, Charlottesville, VA USA

## Abstract

**Background:**

Spousal care partners to people with dementia (PWD) have a higher rate of depression and anxiety when compared to similar age controls. Previous studies have suggested a role of gut microbiota in the pathophysiology of neuropsychiatric symptoms and Alzheimer’s disease (AD). Thus, our study aims to: (1) determine the presence and severity of depression and anxiety in care partners of PWD, and (2) determine the concentrations of short chain fatty acids (SCFA), which are mainly produced by gut microbiota and are important in mediating gut microbiota effects, in the blood of care partners of PWD.

**Methods:**

Three groups of participants were recruited for this cross‐sectional study: healthy controls, patients with AD and their spousal care partners. We used the Beck Depression Inventory (BDI‐II) to assess the presence and severity of depression, the State‐Trait Anxiety Inventory to evaluate care partner distress, and repeatable battery for the assessment of neuropsychological status (RBANS) for cognitive assessment. Their blood and feces were harvested.

**Results:**

We recruited 53 participants: 15, 21 and 17 participants in the control, care partner and AD groups, respectively. Patients with AD had lower mean (SD) total RBANS scores [69 (16)] than controls [110 (11)] (P < 0.001) and care partners [111 (13)] (P < 0.001). Patients with AD and their care partners had higher state and trait anxiety scores than controls (Fig. 1). Care partners had the highest BDI scores (Fig. 2). Care partners had higher concentrations of acetic acid, propionic acid and butyric acid in the blood than those in the control group (Fig. 3).

**Conclusions:**

Spousal care partners had evidence of increased levels of depression, anxiety, and concentrations of SCFAs relative to controls. Our results suggest that care partners to PWDs have gut microbiota different from that of controls, which may be related to their elevated anxiety and depression.